# Evaluation of comparative efficacy of *Celastrus paniculatus (Jyotishmati) *capsule versus sertraline capsule in the management of *Chittodvega* (generalized anxiety disorder): protocol for a randomized controlled trial

**DOI:** 10.12688/f1000research.139473.1

**Published:** 2023-12-11

**Authors:** Reeya Gamne, Sadhana Misar, Mayank Rai

**Affiliations:** 1 Department Kayachikitsa, Mahatma Gandhi Ayurved College Hospital and Research Centre, Datta Meghe Institute of Higher Education and Research, Wardha, Maharashtra, India

**Keywords:** Chittodvega, Celastrus paniculatus, GAD, HAM-A Scale, Jyotishmati, Medhya, Sertralin

## Abstract

**Background:**

Generalized anxiety disorder (GAD) *(Chittodvega)* is one among many types of mental disorders explained in Ayurveda. It can be defined as a *Chitta* (mind) + *Udvega* (anxiety)= *Chittodvega*- Anxious status of a mind. *Celastrus paniculatus* is one of the most important medicinal plants of the Celatraceae family commonly known as *Jyotishmati.* It stimulates and improves the digestive fire and metabolism at a cellular level ( *Jatharagni and Majja dhatwagni).* It can be correlated to GAD. GAD is characterized by feelings of threat, restlessness, irritability, sleep disturbance, and tension, and symptoms such as palpitations, dry mouth, and sweating. It affects women more frequently than men and prevalence rates are high in midlife (prevalence in females over age 35: 10%) and older subjects. In modern medicine the first-line psychological and pharmaceutical treatments are selective serotonin reuptake inhibitors (SSRIs) like sertraline (SNRIs).

**Aim and objectives:**

To evaluate the comparative efficacy of *Jyotishmati* versus sertraline in the management of *Chittodvega.*

**Methods:**

In this randomized clinical equivalence trial a total of 70 patients will be enrolled and divided into two equal groups. Patients between 20–50 years age of either gender having symptoms of *Chittodvega* and a Hamilton anxiety rating (HAM-A) scale score less than 24 (i.e., mild to moderate) will be selected for the study. In Group A, sertraline capsules 25 mg for first 7 days and then dose increased to 50 mg at bedtime for next 53 days and in Group B *Jyotishmati* Capsules 500 mg will be given twice a day after food with water for 60 days.

**Result and observation:**

The patients will be assessed on the HAM-A scale, serum cortisol and WHO Quality of Life on day 0, 30, 60 and 90 and data collected will be analysed by using statistical tests.

**Trial registration:**

CTRI No. REF/2023/07/069880 Date – 15/09/2023

## Introduction

Ayurveda, due to its psychosomatic approach considers the mind as an integral part of life. It advocates the integration between the mind, body and soul with holistic approach. In our bodies, there are two different types of mind qualities ( *Manas doshas* like *Satva*, *Rajas*, and *Tamas*) and bodily humours like ( *Vata, Pitta,* and *Kapha*). Because bodily humours and mind qualities frequently interact with one another, ^1^ Ayurveda accepts the idea of psychosomatic disorder. Of the three humours, *Vata* (Particularly *Prana, Vyana, and Udana Vata*) is primarily in charge of controlling and stimulating mental activity as well as generating enthusiasm. *Sadhaka*, a subtype of *Pitta*, has a direct connection to how the mind works. It is in charge of intelligence, memory, and cognition, as well as self-worth and enthusiasm. ^2^ The qualities of *Kapha* ( *Tarpaka* and *Bodhaka*, types of *Kapha*) include endurance, strength, knowledge, learning, wisdom, thinking, understanding, comprehensiveness, comprehension ability, equilibrium, enthusiasm etc. ^3^ Generalized anxiety disorder (GAD) ( *Chittodvega*) is referred to as the “anxious status of a mind” and is described as a *Chitta* (mind) + *Udvega* (anxiety). ^4^ It is referred to as *Raja-tama vikara* in *Charaka vimana* 6 ( *Rogaanik vimaana adhyaya*). ^5^

The vitiation of mind qualities like *Raja* and *Tama* leads to the development of *Chittodvega.* *Raja* is aggravated by indulgence in causative factors, which also aggravates another mind quality, i.e., *Tama* as well as *Vata*, and *Pitta.* In Ayurveda *Chittodvega* (anxiety disorders) is described as one of the causes of psychosis ( *Unmada)* rather than as a separate disease. ^6^ One of the minor psychiatric diseases is *chittodvega*; there is no disorientation and the patient can carry out everyday activities without much trouble (i.e neurosis), ^7^ whereas psychosis ( *Unmada*) is a major psychological disorder (impairment of orientation, i.e. psychosis), and neurosis may leads to psychosis, so can be considered as prodromal of psychosis ( *Unmada*). ^8^

The symptoms of Chittodvega are similar to those of GAD, which are muscle tension, poor concentration, autonomic arousal, feeling “on edge” or restless, and insomnia. GAD manifests as persistent, excessive, and/or exaggerated worry. The Diagnostic and Statistical Manual of Mental Disorders, Fifth Edition (DSM-5) includes the following diagnostic criteria: anxiety and worry that has lasted at least six months. ^9^ Controlling the worry is difficult. For at least 6 months, the anxiety is connected with three or more of the following symptoms: 1. Restlessness, tenseness, or apprehension. 2. Being easily exhausted. 3. Difficulty concentrating or going blank, as well as impatience. 4. Muscle tenseness. 5. Sleep deprivation. Irritability: anxiety causes severe distress or impairment in social and work settings. ^10^ There is no physiologic reason for the worry. ^11^

Many disorders are more common today, and one of them is mental illness, which stands as India’s third largest health burden according to a World Health Organization (WHO) report. ^12^ WHO estimates that 264 million people globally, or 3.6%, suffer from an anxiety illness. Additionally, anxiety affects 4.6% of females and 2.6% of males worldwide. ^13^ The following primary categories of anxiety disorders are included in the DSM-5, according to the American Psychiatric Association: acute stress disorder, post-traumatic stress disorder, obsessive compulsive disorder (OCD), GAD, agoraphobia without panic, social phobia (social anxiety disorder), specific phobia, and anxiety disorder not otherwise mentioned. ^14^

In modern medicine, GAD management consists of psychotherapy, like cognitive behavioral therapy (CBT) and pharmacological approaches such as cannabis, citalopram, escitalopram, sertraline, duloxetine, and venlafaxine. ^15^ The first-line psychological and pharmaceutical treatments are CBT and selective serotonin reuptake inhibitors (SSRIs) like sertraline; alternative possibilities include selective norepinephrine reuptake inhibitors (SNRIs). Compared to other anxiolytics, SSRIs deal with depression, which is frequently co-occurring with anxiety and has less adverse effects. ^16^ The World Federation of Societies of Biological Psychiatry recommends SSRIs, and SNRIs as first-line therapies for GAD. However the medicine should treat the condition without any adverse effects on the body. So, there is a need to search for better treatment with less complications.

One of the most significant medicinal plants in the Celatraceae family is *Celastrus paniculatus*, also known as *Jyotishmati.* It is a deciduous, woody forest climber that grows primarily in mountainous terrain. *Jyotishmati* is a combination of the words “enlightens” (Jyoti) and “brain functions” ( *mati*), which means “enlightens the psychomotor function.” The seed oil ( *Jyotishmati taila*) is well renowned for its intellect-promoting ( *medhya*) properties. As a brain tonic, it is used to boost intelligence and improve memory. ^17^ With its pungent and bitter taste and hot potency, *Celastrus paniculatus* ( *Jyotishmati*) has an effect on three bodily humours of the body. It strengthens the ability of grasping and remembering (i.e *Grahana* and *Smarana*) and supports *Sadhak Pitta* (a subtype of pitta). Both digestion and cellular metabolism ( *Jatharagni* and *Majja dhatwagni*) are stimulated and improved by its attributes. ^18^ By clearing and opening the micro channel, it enhances circulation and offers appropriate nutrition. ^19^^,^^20^

In *Bhavaprakash Nighantu* and *Raj Nighantu* it is mentioned as intellect promoting ( *Medhya*) drug. ^21^

## Need for the study

One type of anxiety illness, known as generalized anxiety disorder (GAD), is marked by excessive worry and anxiety that the individual with it has trouble controlling. GAD leads to functional disability and a markedly reduced quality of life for the patient. ^22^ This constituted 25–30% of psychiatric outpatients. ^23^ International Statistical Classification of Diseases and Related Health Problems ICD 10 enlists anxiety as one of the symptoms. Anxiety has a significant financial impact on the healthcare system that cannot be overlooked. Its prevalence is about 2–4%, along with psychological arousal, muscle tension, sleep problems, and restlessness. ^24^ Many people live with GAD, which makes it difficult for them to engage in relationships, employment, and other parts of life. Due to the problems of living in a materialistic, competitive society as well as the modern way of life, these types of symptoms and diseases are becoming more and more prevalent day by day. ^25^

To reduce the impact of these ongoing health hazards, treatment regimens are always being sought for the management of anxiety and the reduction of stress. Substitutes to SSRIs, benzodiazepines, and other prescribed medication are highly sought after in an effort to reduce exposure to hazardous effects related to these drugs. ^26^

The intellect promoting ( *Medhya*) medicines described in the Ayurvedic texts have a powerful effect on the mind, have the ability to lower anxiety, and support mental wellness. It is also mentioned in the classics that the one who uses rejuvenating ( *medhya rasayana)* drugs would attain longevity, memory, intellect, youth, luster, skin tone and voice, excellent functioning of the body and sense organs.

Presently there is a need for safer and effective drugs for GAD ( *Chittodvega)* and less research have been done in this regard in recent years. *Celastrus paniculatus* is one such herb mentioned having intellect promoting *(Medhya)* property. ^27^

As a result, this study aims to assess the effectiveness of *Celastrus paniculatus* ( *Jyotishmati)* capsules in the treatment of *Chittodvega.*

**Aim:** Evaluation of comparative efficacy of *Celastrus paniculatus* ( *Jyotishmati)* capsules versus sertraline capsules in the management of *Chittodvega* (generalized anxiety disorder).

### Objective

*Primary*

• To evaluate the efficacy of *Jyotishmati* capsules on the Hamilton anxiety rating-scale (HAM-A) and serum cortisol level.
• To evaluate the efficacy of sertraline capsules on the HAM-A Scale and serum cortisol level.
• To compare the efficacy of both on the HAM-A Scale and serum cortisol level.

*Secondary*

• To evaluate the efficacy of *Jyotishmati* capsules on quality of life.
• To evaluate the efficacy of sertraline capsules on quality of life.
• To compare the efficacy of both on quality of life.

## Methods

### Ethical considerations

The study will start after clearance from the institutional ethics committee of Mahatma Gandhi Ayurved College Hospital and Research Centre, Salod (H), Wardha. And after CTRI registration (Ref. No. REF/2023/07/069880) Date – 15/09/2023.

The committee will decide on the endpoint and oversee the trial as it progresses. The researcher will assess any adverse event and will report these to the ethics committee. The committee will decide on the endpoint and oversee the trial as it progresses.

Before conducting the trial, written informed consent will be taken from the patient in the local language while explaining every aspect of the study. The researcher will take consent from trial participants.

The personal information of the participants will be collected and kept confidential before, during, and after the trial. Physical data will be stored in a protected storage facility with only access to the researcher. Computerized data will be held in a password-protected hard drive with only access to the researcher.

### Study setting

The patients will be selected from Kayachikitsa Out-patient department and In-patient department of the Mahatma Gandhi Ayurved College, Hospital & Research Centre, Salod (H) and peripheral camps, mainly the population of Wardha district including patients of all community, (Maharashtra) India.

A total of 70 patients will be recruited for the study. They will randomly be divided into two groups; Group A – sertraline capsules, and Group B – *Jyotishmati* capsules. All the baseline parameters will be recorded at the start of the study. The patients will undergo treatment for 60 days for both groups. All the parameters will be recorded at the 0 ^th^, 30 ^th^, 60 ^th^, 90 ^th^ day of the study duration.

### Eligibility criteria

*Inclusion criteria*

• Patients willing to take part in a research study and ready to give written informed consent.
• Patients will be selected according to DSM-V, ^28^ ICD-10 ^29^ criteria for anxiety disorders.
• A score of less than 24 on HAM-A scale.
• Age group of 20 to 50 years of either sex.
• Free of psychiatric conditions other than anxiety.

*Exclusion criteria*

• Current primary diagnosis of a comorbid disorder deemed to require treatment prior to GAD, such as major depressive disorder, panic disorder, social phobia, obsessive compulsive disorder, post-traumatic stress disorder, or drug dependency.
• Currently undertaking CBT with a psychologist.
• GAD *(Chittodvega)* due to drug abuse, a medication or due to general medical state like hyperthyroidism.
• Alcoholic, and self-harm or suicide risk.
• Diagnosed with psychosis, schizophrenia, or bipolar illness currently or in the past.
• Treatment with sertraline for two or more weeks within the previous three months.
• Pregnant and lactating mothers.

### Intervention description

• **Group A** – sertraline capsules 25 mg at bedtime for 1 week, then increased to 50 mg with water for 60 days.
• **Group B** – *Jyotishmati* capsules containing processed seed powder of Jyotishmati, 500 mg twice a day with water for 60 days.

*Preparation*

*Jyotishmati (Celastrus paniculatus)* seed will be pulverized into powdered form which then will be sieved and three bhavanas (trituration) of *Jyotishmati phanta* will be given to it according to *Churnakriya* method mentioned in *Sharangadhara Samhita* in Dattatreya Ayurved Rasashala, Salod (H), Wardha. ^30^ The processed powder will be filled into capsules.

*Intervention modification*

Any adverse effect during the treatment will be noted and will be informed to the ethical committee. The patients will be taken care of for the adverse effect. If participants want to drop out, this will be mentioned with the reason for discontinuing the treatment.

### Outcomes

• **Primary –** Reduction in Hamilton A score and serum cortisol levels,
• **Secondary –** Improvement in quality of life.

### Participant timeline

**Figure 1.**
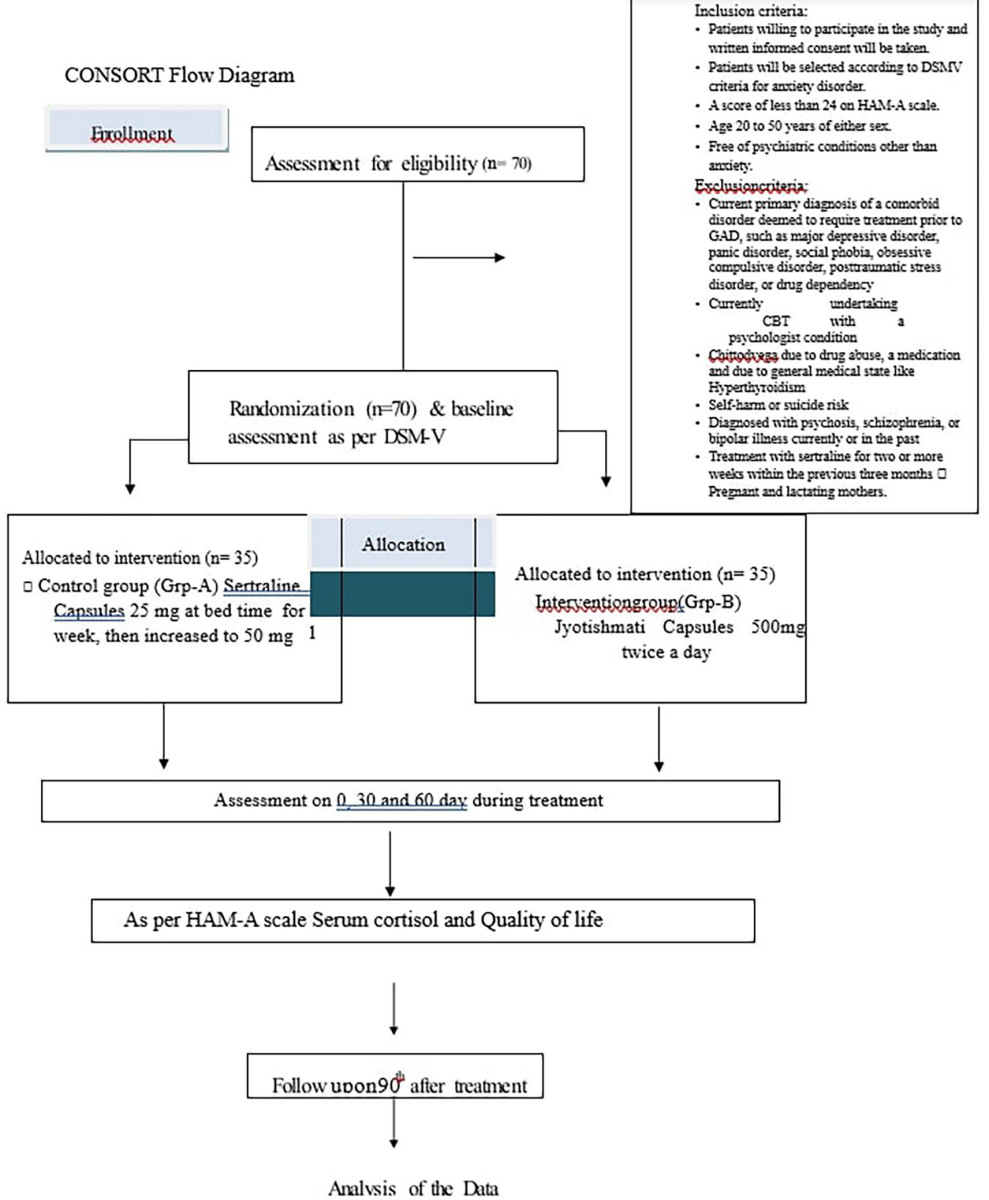
Showing study timeline.

Patients will be treated for 60 days and assessment will be done up to 90 days as mentioned in Figure 1.

### Sample size formula using mean difference

$$n1=n2=2\frac{{\left(Z_{\alpha }+Z_{\beta }\right)}^{2}\sigma^{2}}{{\left(\delta \right)}^{2}}$$

$$Z_{\alpha }=1.96$$

α=Type I error at 5% at both sides two tailed

$$Z_{\beta }=1.28=\text{Power at 90\%}$$

Primary Variable = Hamilton anxiety rating scale (Anxiety Assesment)

Before the treatment (sertraline) Hamilton Anxiety Rating Scale (mean ± sd) = 19.38 ± 4.318, (As per reference article). ^31^

After the treatment (sertraline) Hamilton Anxiety Rating Scale at 28 day (mean ± sd) = 15.70 ± 4.550,

Mean difference (δ) = 3.68

Pooled Std. Deviation = (4.318+ 4.550)/2 = 4.434

Sample size N = 31

Considering 10 % drop out = 3

Total sample size required = 31+3 = 34 per group.

For protocol purposes and according to the calculated sample size for each group is = 34

### Recruitment

As per sample size calculation, total 34 patients will be recruited which include 31 patients and 3 drop outs. Data collected will be analyzed by using appropriate statistical methods. The patients of *Chittodvega* will be selected from the Kayachikitsa Out-patient department and In-patient department of Mahatma Gandhi Ayurved College, Hospital & Research Centre, Salod (H), and from specialized peripheral camps.

A total of 70 patients will be recruited for the study.

### Enrolment and interventions time schedule

The intervention period will be from 0 to 60 days and follow up on the 0 ^th^, 30 ^th^, 60 ^th^ and 90 ^th^ day.

### Guidelines

This protocol follows the SPIRIT guidelines. ^23^

### Case definition

Patients between 20–50 years of age of both gender having symptoms of *Chittodvega* and a HAM-A scale score of less than 24, i.e., mild to moderate cases, will be selected for the study.

### Type of study

Interventional study.

### Study design

Randomized active controlled double blind equivalence trial.

**Investigation** – Serum cortisol level.

**Diagnostic criteria –** DSM-V Diagnostic Criteria for Generalized Anxiety Disorder. ^32^

### Assessment criteria

1. Hamilton Anxiety Scale – HAM-A assessment will be done on 0, 30, 60 and 90 days by asking the validated questionnaire attached in annexure. ^33^
2. Serum cortisol level – will be assessed before and after treatment in our lab.
3. WHO Quality of life – assessment will be done on 0, 30, 60 and 90 days by asking the validated questionnaire attached in annexure. ^34^

### Assignment of interventions

**Allocation sequence generation** – Computer-generated random numbers.

**Allocation implementation** – The researcher or the first author will generate an allocation sequence, enrol participants, and assign participants to intervention.

### Sampling procedure

Randomization will be done by computer-generated random allocation software by SPSS 7.0 to avoid bias in the study.

### Concealment of allocation

A third person will do coding to allocate subjects in sequentially numbered, opaque, sealed envelopes in groups A or B (SNOSE Scheme) to avoid bias in the study.

**Blinding –** Randomized active controlled double blind equivalence trial, i.e., trial participants and care providers will be blinded in the study.

### Data collection plan

Data will be assessed according to HAM-A Scale ***,*** WHO Quality of life, on 0 ^th^, 30 ^th^, 60 ^th^ & 90 ^th^ day and serum cortisol at baseline and after completion of treatment. See Table 1.

**Table 1.** Plan for collection of data.

Group	Sample size	Intervention	Dose and frequency	Anupana	Duration	Follow up
A	35	Sertraline Capsule	25 mg at bed time for 1 week, then increased to 50 mg	With water	60 days	Day 0 ^th^, 30 ^th^, 60 ^th^, 90 ^th^
B	35	*Jyotishmati* Capsule	500 mg twice a day after food	With water	60 days	Day 0 ^th^, 30 ^th^, 60 ^th^, 90 ^th^

### Drug collection/authentication

The raw material for the drug will be purchased from an authentic shop from Nagpur (Wagh brothers) and will be identified by the department of *Dravyaguna* and *Rasashastra* of M.G.A.C.H. and RC, Salod, Wardha.

### Data analysis

After the study, the data will be analyzed according to a suitable statistical test.

The collected data will be entered in the software SPSS version 22. Man-Whitney and Wilcoxon tests will be used if the distribution of data was not normal. A chi squared test will be used to compare the qualitative variables. Independent t-test will be used to compare the mean of the two groups in the normal distribution of data, and for comparison before and after data, the paired t-test will be used.

### Study status

Drug preparation is in process.

## Discussion

*Chitta* (mind) + *Udvega* (anxiety) = *Chittodvega*, or anxious mental state. However, *Chittodvega* is a more appropriate expression for describing the entire anxious illness. ^35^ As a result, the term *Chittodvega* is compared to GAD in this study. GAD patients exhibit chronic, excessive, and/or unreasonable worry that is accompanied by muscle tension, reduced focus, autonomic arousal, a feeling of being on edge or restless, and insomnia for at least six months. This disease causes the patient to have unfocused worry and anxiety that is not related to recent traumatic experiences, but it may be worsened by specific circumstances. ^36^ GAD is a common mental condition in the general society as a whole with a prevalence of about 5%. Women are twice as likely as men to have this condition. ^37^ Medication such as selective serotonin reuptake or serotonin-norepinephrine reuptake inhibitors are the first-line treatments ^38^ but long-term use can have negative side effects such nausea, trouble sleeping, changes in appetite, dry mouth, headaches, and sexual dysfunction. ^39^ In contrast, Ayurveda states that mental disorders ( *manas rogas)* is where mind promoting ( *Medhya*) medicines are used. *Jyotishmati* ( *Celastrus paniculatus*) is a thorny climbing polygamodioecious shrub that may climb up to 10 m with the help of a nearby tree. It is found virtually across India, rising to an elevation of 1800 m in the subtropical Himalayas. ^40^ The seed oil and fruit are often used for their tranquillizing, sedative, wound-healing, and other properties. The Ayurvedic application is primarily for its Medhya (brain tonic) activity. It can be used as a brain tonic to enhance intelligence and memory. Its *Deepana* (stimulating digestion) property help in improving deranged digestive fire ( *Agni*) and thereby balancing bodily as well as mental humours ( *Sharir* and *Manas dosha*) ^41^ *Jyotishmati* is a renowned medicinal herb recognised for its brain-boosting properties that has been used for generations in Ayurvedic medicine to sharpen memory and improve concentration and cognitive function. ^42^

**Data monitoring:** formal committee.

### Dissemination

This protocol will be further published as a thesis to disseminate the study for GAD ( *Chittodvega*). The study protocol provides a detailed overview of the study design, methodology, data collection procedures, data analysis plan, and ethical considerations. By disseminating this protocol, we hope to advance knowledge in the field and facilitate future research by posters, papers and publications.

## Data Availability

**Zenodo:** SPIRIT checklist for ‘Evaluation of comparative efficacy of *Celastrus paniculatus (Jyotishmati)* capsule versus sertraline capsule in the management Of *Chittodvega* (generalized anxiety disorder) – protocol for a randomized controlled trial’. https://doi.org/10.5281/zenodo.8125916. ^43^ Data are available under the terms of the Creative Commons Attribution 4.0 International license (CC-BY 4.0).
