# Supplementary material for: Evaluation of comparative efficacy of Celastrus paniculatus (Jyotishmati) capsule versus sertraline capsule in the management of Chittodvega (generalized anxiety disorder): protocol for a randomized controlled trial
Source: F1000Res. 2024 Oct 28;12:1577. Originally published 2023 Dec 11. [Version 3] doi: 10.12688/f1000research.139473.3 (PMC11305454; doi:10.12688/f1000research.139473.3)
Supplement: Peer review history

# Reviewer report: Reviewer response for version 3

**DOI:** 10.5256/f1000research.173733.r349444

**Published:** 2025-01-16

**Authors:** Rachael Frost

**Affiliations:** 1 Liverpool John Moores University, Liverpool, England, UK

This manuscript outlines a trial protocol comparing *Celastrus paniculatus *with sertraline. The study is a valuable one and will contribute to our understanding of plant extracts for anxiety. However, the protocol needs to be more clearly reported, with greater contextual information and consider particularly the issue of blinding and the type of analysis (see detailed points below).

 Abstract:

- The information about GAD should come before Celastrus info.
- Why is SNRI in brackets at the end of the background? Sertraline is SSRI not SNRI.
- Background section should briefly summarise if previous studies have found positive or null results.
- Primary outcome should be stated in results section.
- A statement regarding the impact of the results should be included.

Introduction

- Good explanation of how Ayurvedic concepts relate to GAD and therefore the rationale for choice of plant to test
- I am not aware of cannabis commonly being used as a GAD treatment (I am in the UK and certainly it is not the case here, but I am not sure elsewhere) – CBD extract may be more commonly used over the counter if that is what the authors are referring to?

- “International Statistical Classification of Diseases and Related Health Problems ICD 10 enlists anxiety as one of the symptoms.” – symptoms of what?
- “less research have been done in this regard in recent years” – a review we carried out showed an increase over time in herbal anxiety research, albeit still at much smaller numbers than depression.
- There is no discussion of any prior Celastrus research regarding anxiety or other mental disorders. Please include this, or a statement that there is none. It is important to contextualise the current study.  
- Objectives are phrased somewhat unusually – most trials simply state comparative objectives without additional within-group change objectives (which are not that useful).

Methods:

- The committee will decide on the endpoint and oversee the trial as it progresses – sentence is repeated twice. Plus this seems unusual – studies should have a predefined endpoint as part of the protocol, with a committee deciding on early termination if needed.
- Ethical considerations should include withdrawal.
- “Peripheral camps” – not sure what the authors mean here, has something been lost in translation?
- Age group inclusion criteria needs justification. Why do the authors not use a minimum scale cutoff of HAM-A baseline? There is no mention of chittodvega, are the authors not measuring/assessing this or assuming it is synonymous with GAD? Otherwise inclusion and exclusion criteria are reasonable.
- Trial is described earlier as double blind, but the capsules are taken at different times (bedtime vs twice a day) – this would seem to unblind participants?
- Drug collection should be grouped with preparation, and all details in herbal CONSORT checklist (Gagnier et al, 2006) should be followed as there are insufficient details at present (e.g. voucher specimen)
- Authors should specify whether adverse effects will be asked about in a structured way or will rely on participants’ spontaneous reporting.
- It is unclear why there are two primary outcomes, this is usually not recommended. The study is powered on HAM-A scores indicating this is the primary outcome.
- Figure 1 should be clearer and red spell check underlines removed from text boxes.
- The methods needs to be grouped more logically, similar information is presented in different places with lots of headings with little information underneath. This makes it hard for the reader to absorb relevant details. The assessment criteria presents the outcomes – this should be moved under outcomes to more clearly present what measures are being used. Likewise data analysis is discussed under recruitment, when there is already a separate section for analysis. Diagnostic criteria should go with inclusion criteria and Investigation is an outcome.
- Recruitment section should specify 34 patients per group as it currently says in total at the start of this section.
- Allocation implementation – The authors have contradictory statements on allocation implementation and concealment – one says it will be done by the same person, another by a third person. Please ensure consistency and write relevant information under the same heading in the same place.
- Under sampling procedure the authors discuss generation of allocation sequence. Allocation is not sampling, this belongs under the sequence generation heading above.
- Blinding – as above, if capsules are taken at different times in different groups, this leads to a high risk of unblinding. It needs to be clear whether the clinician/researcher assessing HAM-A scores at the end of the trial is blinded, as this is the largest influence on results.
- Table 1 plan for collection of data does not include the measures collected at each timepoint but focuses on interventions. I suggest the authors base this on the SPIRIT example.
- Analysis – tests are generally appropriate but it is unclear what chi-squared would be used for as all variables appear to be continuous. If groups are to be compared at baseline or vs dropouts it should state this. Analysis type (ITT, PPA, complete case etc) should be stated, with how missing data will be handled. Authors should also ensure that those withdrawing from the intervention (e.g. due to side effects) remain in the trial for collection of outcome data to reduce bias. Please specify if analyses will be adjusted for any factors.
- Compliance is not discussed. Authors should state how compliance will be measured.
- Data monitoring belongs under methods not discussion as per SPIRIT. More detail than ‘formal committee’ is required.

Discussion:

- Very limited and mainly repeats the introduction. The authors should outline the strengths and limitations of the proposed trial, and expand upon the potential implications for research and practice.
- Dissemination should include where the results, not just the protocol, will be disseminated.

Is the study design appropriate for the research question?

Yes

Is the rationale for, and objectives of, the study clearly described?

Partly

Are sufficient details of the methods provided to allow replication by others?

Partly

Are the datasets clearly presented in a useable and accessible format?

Not applicable

Reviewer Expertise:

Ageing, mental health, herbal medicines, behavioural interventions

I confirm that I have read this submission and believe that I have an appropriate level of expertise to confirm that it is of an acceptable scientific standard, however I have significant reservations, as outlined above.

# Reviewer report: Reviewer response for version 2

**DOI:** 10.5256/f1000research.167827.r306252

**Published:** 2024-08-05

**Authors:** Danish Javed

**Affiliations:** 1 All India Institute of Medical Sciences, Bhopal, India

**Introduction: **I have thoroughly reviewed the article. The review was conducted with a focus on the relevance, accuracy, and overall contribution of the content to the field of Ayurveda.

**Content Analysis:**

1. **Relevance:**
  The article is highly relevant to the current trends and practices in Ayurveda. It addresses significant issues and presents well-researched information that aligns with contemporary medical knowledge and practices.
2. **Accuracy:**
  The information presented in the article is accurate and supported by credible references. The data and facts have been cross-verified with existing literature, ensuring the reliability of the content.
3. **Clarity and Coherence:**
  The article is well-written, with clear and coherent language. The flow of information is logical, making it easy for the reader to follow and understand the concepts discussed.
4. **Contribution to the Field:**
  This article makes a valuable contribution to the field of Ayurveda. It provides new insights and practical approaches that can be beneficial for practitioners and researchers alike.
5. **Methodology:**
  The research methodology adopted in the article is robust and appropriate for the study. The methods used are well-explained and justified, adding to the credibility of the findings.
6. **References and Citations:**
  The article includes a comprehensive list of references and citations from reputable sources. This enhances the article's scholarly value and allows readers to further explore the topics discussed.

Based on my thorough review, I have found the article to be suitable for index. It meets the high standards required for scholarly work in the field of Ayurveda. The content is relevant, accurate, and contributes significantly to the existing body of knowledge. Therefore, I recommend this article for indexing without any reservations.

Is the study design appropriate for the research question?

Yes

Is the rationale for, and objectives of, the study clearly described?

Yes

Are sufficient details of the methods provided to allow replication by others?

Yes

Are the datasets clearly presented in a useable and accessible format?

Yes

Reviewer Expertise:

Ayurveda

I confirm that I have read this submission and believe that I have an appropriate level of expertise to confirm that it is of an acceptable scientific standard.

# Reviewer report: Reviewer response for version 2

**DOI:** 10.5256/f1000research.167827.r291859

**Published:** 2024-07-16

**Authors:** Punam Khobarkar

**Affiliations:** 1 Kayachikitsa, All India Institute of Ayurveda, New Delhi, India

Dear Authors

 You have addressed the previous suggestions effectively. The introduction is now clearer, the methodology is well-detailed, and the discussion is more comprehensive

 The manuscript has been significantly improved in terms of clarity, consistency, and comprehensiveness. Therefore, I recommend the acceptance of this manuscript for indexing.

Is the study design appropriate for the research question?

Yes

Is the rationale for, and objectives of, the study clearly described?

Yes

Are sufficient details of the methods provided to allow replication by others?

No

Are the datasets clearly presented in a useable and accessible format?

Partly

Reviewer Expertise:

ayurveda cardiology, diabetes, insomnia, herbsl medicine, ayurveda

I confirm that I have read this submission and believe that I have an appropriate level of expertise to confirm that it is of an acceptable scientific standard.

# Reviewer report: Reviewer response for version 1

**DOI:** 10.5256/f1000research.152749.r286303

**Published:** 2024-06-06

**Authors:** Punam Khobarkar

**Affiliations:** 1 Kayachikitsa, All India Institute of Ayurveda, New Delhi, India

**1.Title:**

  It is precise and informative, giving a clear indication of the research topic.

**2.Abstract:**

 The abstract succinctly summarizes the background, objectives, methodology, and anticipated outcomes. However, it could benefit from a more detailed description of the study design, including specific statistical methods and anticipated sample size, to better inform the reader about the study's robustness and scope.

**3.Introduction**

 The introduction provides a comprehensive overview of GAD, detailing its prevalence, symptoms, and the limitations of current pharmacological treatments, such as side effects and dependency issues. The rationale for investigating Celastrus paniculatus (Jyotishmati) is well-grounded, citing its historical use in Ayurvedic medicine and previous studies suggesting its anxiolytic properties.

**4.Literature Review:**

 The introduction could be strengthened by including a more exhaustive review of contemporary studies and meta-analyses on the efficacy of Jyotishmati and Sertraline, thereby situating the current research within the broader scientific context.

**5.Aims and Objectives**

 The study’s aims and objectives are clearly articulated. The primary objective is to compare the efficacy of Jyotishmati and Sertraline in reducing anxiety symptoms as measured by the Hamilton Anxiety Rating Scale (HAM-A). Secondary objectives include assessing changes in serum cortisol levels and evaluating quality of life improvements.

**6.Methods**

**a.Study Design:**

 The study employs a randomized clinical equivalence trial design, which is appropriate for comparing the effectiveness of two interventions. However, further elaboration on the randomization process, blinding procedures (if any), and controls to mitigate bias would enhance the methodology's rigor.

**b.Participants:**

 Inclusion and exclusion criteria are well-defined, ensuring a relevant study population. Yet, additional details on the demographic characteristics of the target population would be beneficial.

**c.Intervention:**

 The interventions are described with adequate detail, specifying the dosage and duration of treatment for both Jyotishmati and Sertraline. However, the manuscript should provide more information on the standardization and quality control of the Jyotishmati capsules used.

**d.Outcome Measures**:

 Primary and secondary outcome measures are appropriate and clearly defined. The choice of HAM-A for primary outcome and serum cortisol levels, along with quality of life assessments, as secondary outcomes are justified. Including information on the validity and reliability of these measures within the study context would be advantageous.

**e.Data Collection and Analysis:**

 The data collection methods are thorough, but the statistical analysis section is underdeveloped. A more detailed plan, including sample size calculation, power analysis, handling of missing data, and specific statistical tests to be used, would enhance the study’s scientific rigor.

**f. Ethical Considerations**

 The manuscript adequately addresses ethical considerations, mentioning informed consent, confidentiality, and the approval by an institutional ethics committee. This reflects adherence to ethical standards in clinical research.

**7.Expected Outcomes:**

 While the manuscript outlines expected outcomes, it should also discuss potential challenges and limitations in greater detail, such as sample size constraints, adherence issues, and potential biases.

**8.Discussion:**

 The discussion should provide a balanced view of the study's potential impact on clinical practice and future research. It should also elaborate on how the findings could influence the treatment paradigm for GAD, especially in contexts where traditional and alternative medicine practices intersect.

 9. **Strengths:**

 Comprehensive background and rationale for the study.

 Clearly defined aims and objectives.

 Detailed description of interventions and outcome measures.

 Ethical considerations are well addressed.

**10.Weaknesses:**

 The abstract could be more detailed regarding the study design and statistical methods.

 The methodology section lacks comprehensive details on randomization, blinding, and statistical analysis.

 The literature review in the introduction could be more exhaustive.

 The discussion does not sufficiently address potential limitations and the broader impact of the study findings.

 The manuscript presents a well-conceived study protocol to compare the efficacy of Jyotishmati and Sertraline in managing GAD. Addressing the noted weaknesses, particularly in the methodological and analytical details, will enhance the study's scientific validity and reliability. This research has the potential to provide significant insights into alternative treatments for GAD, contributing to the broader field of integrative medicine.

Is the study design appropriate for the research question?

Yes

Is the rationale for, and objectives of, the study clearly described?

Yes

Are sufficient details of the methods provided to allow replication by others?

No

Are the datasets clearly presented in a useable and accessible format?

Partly

Reviewer Expertise:

ayurveda cardiology, diabetes, insomnia, herbsl medicine, ayurveda

I confirm that I have read this submission and believe that I have an appropriate level of expertise to confirm that it is of an acceptable scientific standard, however I have significant reservations, as outlined above.

# Response

**Published:** 2024-06-07

**Authors:** Reeya Gamne

**Affiliations:** Kayachikitsa, Datta Meghe Institute of Higher education, Wardha, Maharashtra, India

THANKS FOR THE REVIEW.

# Reviewer report: Reviewer response for version 1

**DOI:** 10.5256/f1000research.152749.r281408

**Published:** 2024-06-05

**Authors:** Dr Savitha H P

**Affiliations:** 1 Kayachikitsa and manasaroga, SDM College of Ayurveda & Hospital, Hassan, Karnataka, India

1. Evaluation of comparative efficacy of ***Jyotishmati ( Celastrus paniculatus )*** capsule versus sertraline capsule in the management of *Chittodvega* (generalized anxiety disorder) - **A** **Proposed** protocol for a randomized controlled trial

 2. Introduction and need for study - need to  include explanation behind comparing with sertraline as control as there are many other SSRIs. 

 3. Introduction is very lengthy and it needs to be crisp and clear

 4. Repetitions in inclusion and exclusion criteria

 5. Justify about the dose of both control and study drugs

 6. There is difference in the prescription of control drug in abstract and study plan which needs to be corrected

 7. Inclusion of a modern psychiatrist in the study enhances the quality of proposal

 7. Discussion has to be crisp

Is the study design appropriate for the research question?

Yes

Is the rationale for, and objectives of, the study clearly described?

Partly

Are sufficient details of the methods provided to allow replication by others?

Partly

Are the datasets clearly presented in a useable and accessible format?

Not applicable

Reviewer Expertise:

Clinical research, Survey study, Literary research

I confirm that I have read this submission and believe that I have an appropriate level of expertise to confirm that it is of an acceptable scientific standard, however I have significant reservations, as outlined above.

# Response

**Published:** 2024-06-05

**Authors:** Reeya Gamne

**Affiliations:** Kayachikitsa, Datta Meghe Institute of Higher education, Wardha, Maharashtra, India

Thanks for the review.
